# The identity of *Hypolepis robusta*, as a new synonym of *Hypolepis alpina* (Dennstaedtiaceae), based on morphology and DNA barcoding and the new distribution

**DOI:** 10.3897/phytokeys.96.23470

**Published:** 2018-03-15

**Authors:** Morigengaowa, Jun-Jie Luo, Ralf Knapp, Hong-Jin Wei, Bao-Dong Liu, Yue-Hong Yan, Hui Shang

**Affiliations:** 1 Harbin Normal University, Key Laboratory of Plant Biology, College of Heilongjiang Province, Harbin 150025, China; 2 Shanghai Chenshan Plant Science Research Centre, Chinese Academy of Sciences; Chenshan Botanical Garden, Shanghai 201602, China; 3 College of Life and Environmental Sciences, Shanghai Normal University, Shanghai 200234, China; 4 Correspondent of the Muséum National d’Histoire Naturelle (MNHN, Paris, France), Steigestrasse 78, 69412 Eberbach, Germany

**Keywords:** *Hypolepis alpina*, molecular phylogenetic, synonym, taxonomy, Type material

## Abstract

Based on field observations and examinations of herbarium specimens (including type material), consulting the original literature and molecular phylogenetic analysis of the *rbcL* and *trnL-F* sequences, it is concluded that *Hypolepis robusta* is conspecific with *Hypolepis alpina* and is here formally treated as a synonym of it. Additionally *H. alpina* is reported with new distribution records in Guangdong, Guangxi and the Hainan Island of China, respectively.

## Introduction

*Hypolepis* Bernh. (1805) is one of the largest genera in the family Dennstaedtiaceae, with approximately 80 species (PPG I 2016) widespread in tropical and southern temperate parts the world, mainly in tropical Asia and tropical America, but the exact number of species in China is still unclear (Brownsey 1987, Ching 1959, Xing et al. 2013). Amongst them, *Hypolepis alpina* (Blume) Hook. was initially described as *Cheilanthes alpina* Blume from Java in the first publication relating to the ferns of Malaya (Blume 1828). It was later transferred to *Hypolepis* by Hooker (1858) in the last comprehensive treatment of the genus (Brownsey 1987). Afterwards, one endemic species in the Taiwan province of China, *Hypolepis alte-gracillima* Hayata (1915), was reduced to a synonymy of *H. alpina*, according to the *Flora of Taiwan* (Shieh 1975). In addition to Taiwan, *H. alpina* is also distributed in Indonesia, Japan, Malaysia, Papua New Guinea and Philippines (Brownsey 1987, Fig. 1). Subsequently, the species (as *H. alte-gracillima*) was found in Gongshan County, in the Yunnan Province of China and recorded in *Flora Yunnanica* (Chu et al. 2006) as having a Yunnan-Taiwan discontinuous distribution. Another endemic species, *H. robusta* W. M. Chu was described for Yunnan (Chu et al. 2006). This name was treated as a synonymy of *H. polypodioides* (Blume) Hook. (Fraser-Jenkins 2008). Xing et al. (2013) cited a null name, (“*H. robusta* Hayata”) as a synonym of *H. polypodioides* in *Flora of China*, but Hayata’s name has not nomenclatural bearings nor taxonomic implications for Chu’s name. However, even Chu’s *H. robusta* is easily distinguishable from *H. polypodioides* in morphology as an obviously different species. *Hypolepis robusta* has densely multicellular brown glandular hairs and sori protected by well-developed reflexed adaxial indusium, whereas *H. polypodioides* has abundantly colourless non-glandular hairs and sori unprotected or occasionally protected by slightly reflexed green lamina segments. In June 2017, as part of the floristic inventory of Yunnan, *H. robusta* was collected at its type locality, Fugong County and *H. alpina* was collected at its recorded locality in Gongshan County. In addition, during the field work from 2013 to 2017, several specimens of *H. alpina* were collected from Taiwan as well as several others that were initially identified as *H. robusta* in Guangdong, Guangxi, Hainan Island and other locations of Yunnan. After conducting field observations, examinations of the herbarium specimens (including both types studied) and consulting the original literature (Hooker 1858, Chu 1992), it was suspected that *H. robusta* is conspecific with *H. alpina*. Therefore, the identity of *H. robusta* was determined by a more detailed examination of the morphology and molecular phylogenetic analysis.

**Figure 1.**
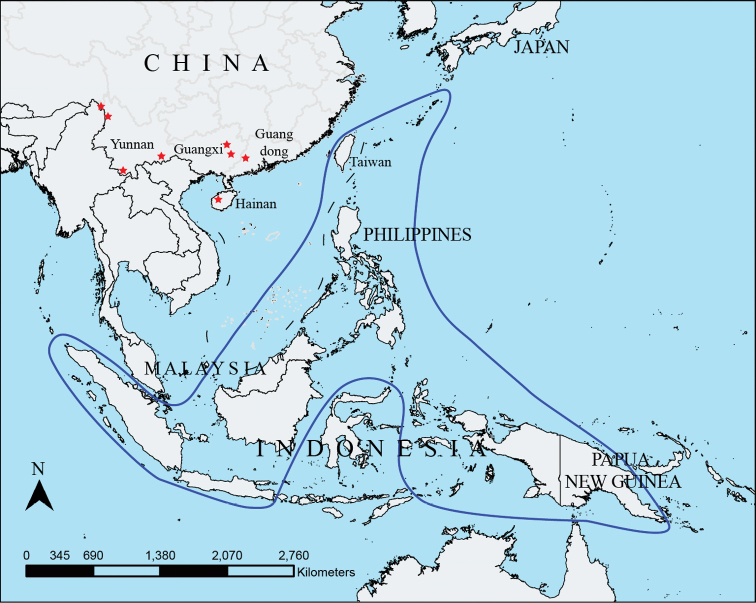
The distributions of *Hypolepis alpina* noted by Brownsey (1987, blue line) and new record localities since then (red stars), using a map available from http://219.238.166.215/mcp/index.asp.

## Materials and methods

### Morphological studies

For morphological comparisons, herbarium specimens or high-resolution images of specimens in CSH, K, KUN, L, P, PE, PYU, TAI, TAIF and US were critically checked. Field observations and collections were made in Guangdong, Guangxi, Hainan Island, Taiwan and Yunnan of China (Suppl. material 1: Table S1).

### Molecular phylogenetic studies

Nineteen specimens were sampled, including the outgroup taxa *Blotiella stipitata* (Alston) Faden and *Histiopteris incisa* (Thunb.) J. Sm., Pteridium aquilinum subsp. wightianum (J. Agardh) W.C. Shieh. Total genomic DNA was extracted from silica gel-dried leaves by using a DNA secure Plant Kit (Tiangen Biotech, Beijing, China) according to the manufacturer’s protocols. The PCR reactions were performed in a Veriti 96-Well Thermal Cycler. Two plastid markers were amplified, the *rbcL* gene and the *trnL-trnF* intergenic spacer. Primers used for amplification and sequencing were: *rbcL* primers 1379R and 1F (Little and Barrington 2003) and *trnL-F* primers trn-F and trn-r1 (Taberlet et al. 1991, Li et al. 2011). The amplification profiles were: initial denaturation (94 °C, 3 min) followed by 29 cycles of amplification, hybridisation and extension (94 °C, 45 s; 52 °C, 30 s; 72 °C, 1.5 min) and 10 min of final extension at 72 °C for *rbcL*, initial denaturation (95 °C, 3 min) followed by 35 cycles of amplification, hybridisation and extension (95 °C, 30 s; 52 °C, 30 s; 72 °C, 1 min) and 10 min of final extension at 72 °C for *trnL-trnF*. Sequencing was conducted using an ABI 3730xl DNA analyser (Applied Biosystems, Invitrogen, Foster City, CA, USA).

### Phylogenetic analyses

Sequences were assembled and edited with SeqMan (DNA STAR package; DNA StarInc., Madison, WI, USA), aligned by Bio Edit (Hall 1999) and adjusted manually where necessary. All sequences are available from GenBank (Table 1).

**Table 1.** Plant materials, voucher information, and GenBank accession numbers of the samples used in the phylogenetic analyses.^a^

Taxon	Voucher	Locality	Geographic coordinates	GenBank accession number	
				*rbcL*	*trnL-F*
*Hypolepis glandulifera* Brownsey & Chinnock	BLD01	Bali, Indonesia	NA	MG944782	MG944788
*Hypolepis robusta* W.M. Chu	DRS005	Darong Mountain, Guangxi, China	NA	MG944773	MG944789
*Hypolepis punctata* (Thunb.) Mett. ex Kuhn	FLX6	Hunan, China	NA	MG944784	MG944790
*Hypolepis tenuifolia* (G. Forst.) Bernh.	HN31	Wuzhishan Mountain, Hainan, China	18°55'1"N, 109°42'13"E	MG944786	MG944791
*Hypolepis robusta* W.M. Chu	HND6	Bawang Mountain, Hainan, China	19°07'26"N, 109°04'46"E	MG944774	MG944792
*Hypolepis alpina* (Blume) Hook.	Knapp4486	Yilan County, Taiwan, China	24°49'N, 121°41'E	MG944769	MG944794
*Hypolepis robusta* W.M. Chu	SG958	Shengtang Mountain, Guangxi, China	NA	MG944777	MG944801
*Blotiella stipitata* (Alston) Faden	SG1185	Kenya	NA	MG944780	MG944795
Pteridium aquilinum subsp. wightianum (J. Agardh) W.C. Shieh	SG1760	Yunnan, China	NA	MG944787	MG944796
*Hypolepis robusta* W.M. Chu	SG1812	Ada Village, Fugong County, Yunnan, China	26°49'5.6964"N, 98°53'36.715"E	MG944776	MG944797
*Hypolepis alpina* (Blume) Hook.	SG1838	Dulongjiang Village, Gongshan County, Yunnan, China	27°41'11.004"N, 98°16'54.340"E	MG944771	MG944798
*Hypolepis alpina* (Blume) Hook.	SG1871	Dulongjiang Village, Gongshan County, Yunnan, China	27°54'49.306"N, 98°20'37.03"E	MG944772	MG944799
*Hypolepis resistens* (Kunze) Hook.	SG2900	Bawangling Mountain, Hainan, China	19°5'28"N, 109°10'59"E	MG944785	MG944800
*Hypolepis polypodioides* (Blume) Hook.	SIWS28	Sulawesi, Indonesia	NA	MG944783	MG944802
*Histiopteris incisa* (Thunb.) J. Sm.	WYD016	Guangdong, China	NA	MG944781	MG944804
*Hypolepis robusta* W.M. Chu	WYD574	Dawu Mountain, Guangdong, China	NA	MG944778	MG944805
*Hypolepis alpina* (Blume) Hook.	YYH11628	Xitou Village, Nantou County, Taiwan, China	NA	MG944770	MG944803
*Hypolepis robusta* W.M. Chu	YYH12064	Mengsong Village, Jinghong City, Yunnan, China	NA	MG944775	MG944793
*Hypolepis robusta* W.M. Chu	ZXC8465	Gulinqing Village, Maguan County, Yunnan, China	22°51'43.64"N, 104°0'15.59"E	MG944779	MG944806

Note: NA = not available. a: Specimens are deposited at the Shanghai Chenshan Botanical Garden Herbarium (CSH), except for voucher Knapp 4486, which is deposited at the Muséum National d'Histoire Naturelle (P).

For phylogeny reconstructions, two methods were used, maximum likelihood (ML) and Bayesian Inference (BI). The ML analyses were conducted with RAxML-HPC BlackBox8.2.10 (Stamatakis 2014). For the Bayesian analyses, the best-fitting models (HKY+G) were selected using jModeltest2 web server under the Bayesian Information Criterion (BIC) (Darriba et al. 2012). Four chains were used with random initial trees as BI settings. Trees were generated for 1,000,000 generations and sampling was conducted every 100 generations. Before stationarity was conducted, the first 2,500 trees were discarded as burn-in trees and the remaining trees were used to construct the majority-rule consensus trees. The remaining trees were used to construct a consensus tree. ML bootstrap values and BI posterior probabilities were labelled on the tree branches.

### DNA barcoding analyses

For species delimitation between *H. alpina* and the other species of *Hypolepis*, the DNA barcoding gap method, based on the Kimura two parameter (K2P) distance, was used. Intra- and inter-taxa genetic distances were evaluated using MEGA 5.0 (Tamura et al. 2011).

## Results

**Figure 2.**
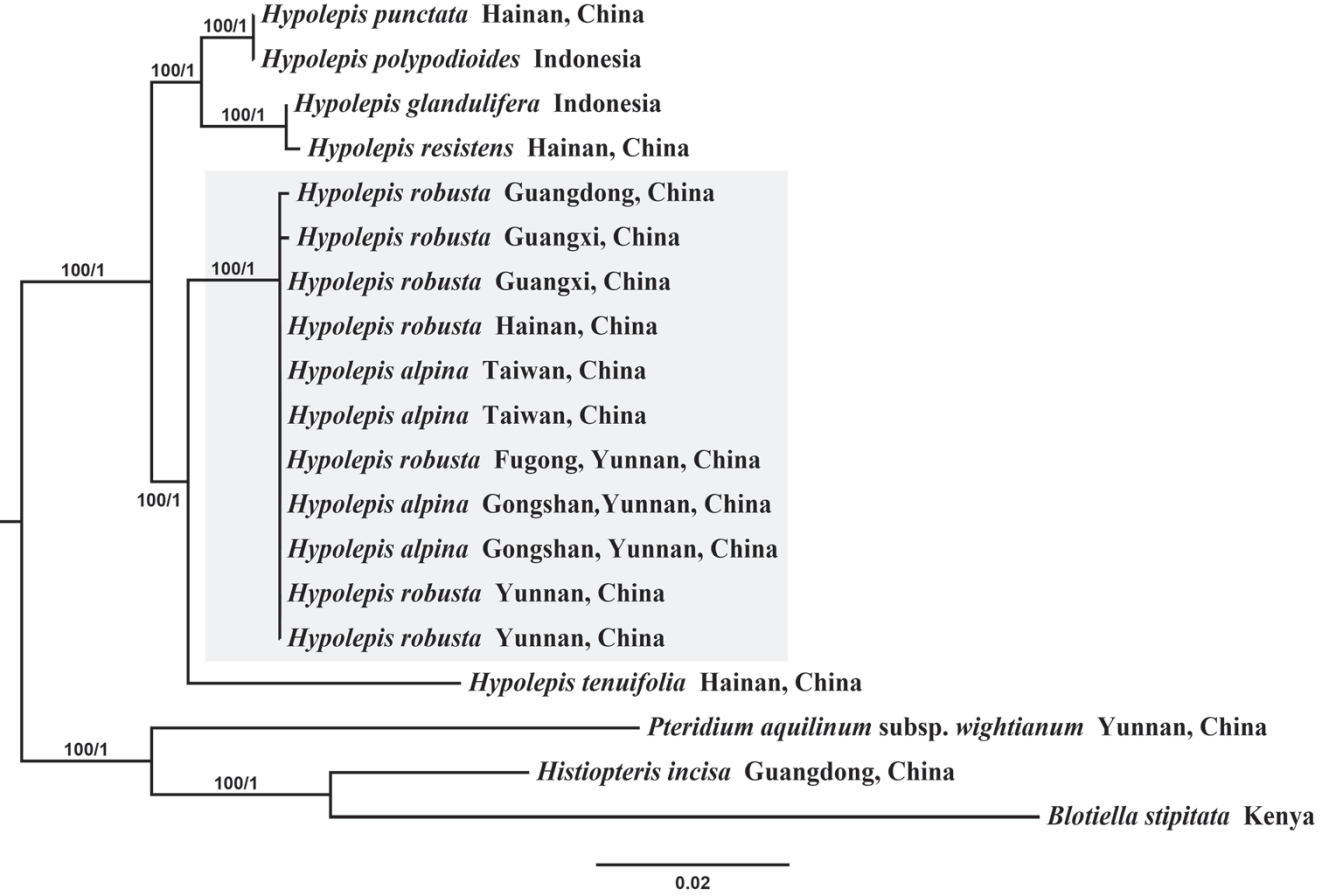
Phylogeny of 16 *Hypolepis* samples and *Blotiella stipitata*, *Histiopteris incisa*, and Pteridium aquilinum subsp. wightianum based on *rbcL* and *trnL-F*. Bootstrap values and Bayesian posterior probabilities are shown along branches (ML/BI).

A total of 19 new sequences amongst the total of 19 specimens were generated in the cpDNA matrix of *rbcL* and *trnL-F* containing 2,166 bp characters with 374 variable sites and 149 parsimony-informative sites. The optimal ML tree showed a negative log-likelihood score (-lnL) of 5577.824547 and the Bayesian tree was consistent with the ML tree. The statistical support is shown along the branches (ML/BI). Individuals of *H. alpina* and *H. robusta* formed a highly supported monophyletic group with an MLBS of 100 as sister clades of *H. tenuifolia*. Moreover, all *rbcL* and *trnL-F* sequences of the *H. robusta*, from type locality, were identical to those of *H. alpina* from Taiwan. The sequences of *H. robusta* from Guangdong, Guangxi and from Hainan Island were also clustered in the *H. alpina* clade, which had an MLBS of 100 (Fig. 2).

**Figure 3.**
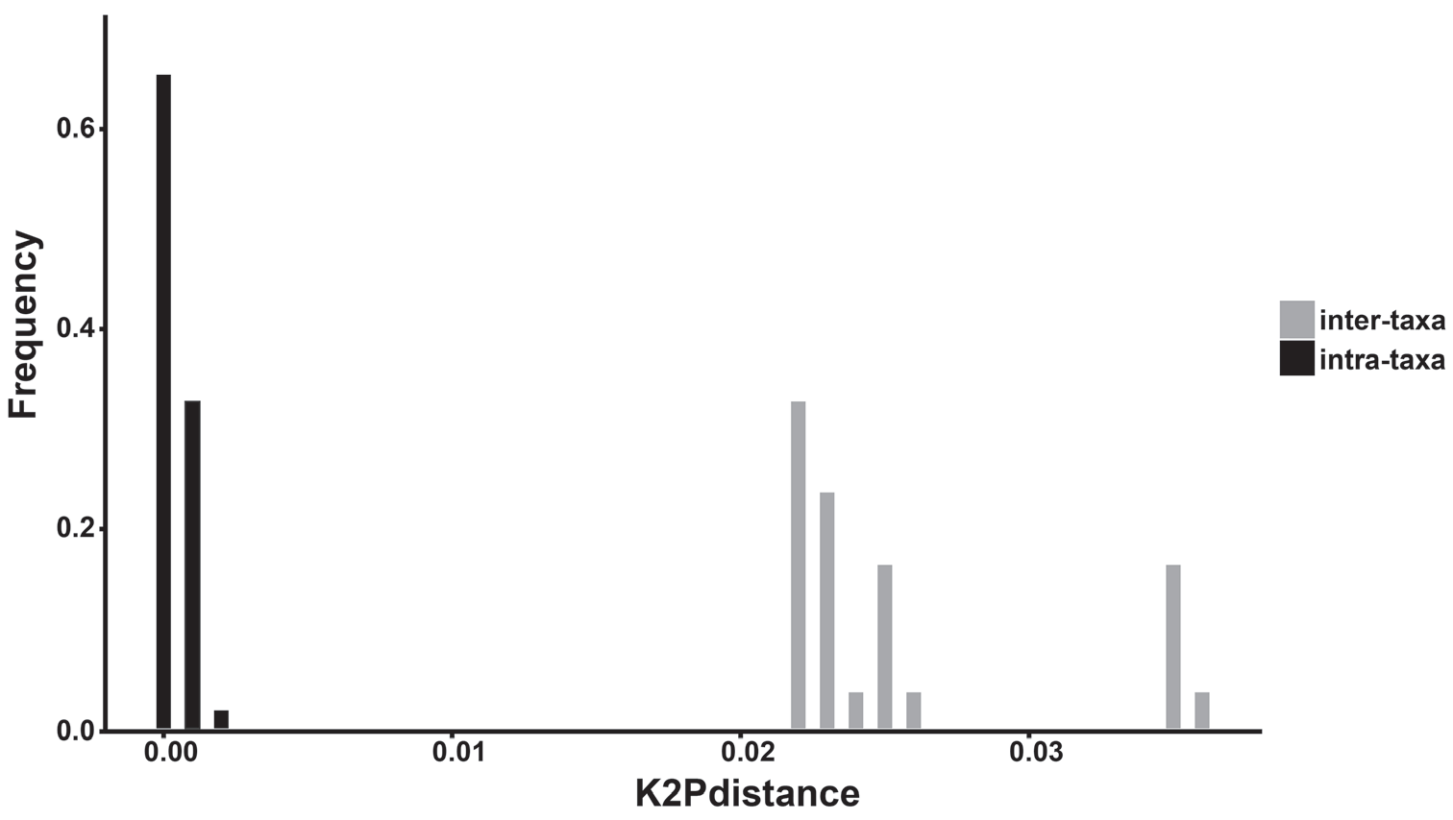
Distribution of intra-taxa (black) and inter-taxa (grey) Kimura two parameter (K2P) distances based on *rbcL* and *trnL-F* sequences as barcode. *Hypolepis alpina* and *Hypolepis robusta* versus the other species of *Hypolepis*.

No differences were observed in the *rbcL* and *trnL-F* barcoding sequences of both *H. alpina* and *H. robusta*, except that two specimens have two base differences respectively. The genetic distance between *H. robusta* and *H. alpina* ranges from zero to 0.002. Their inter-taxon distances were significantly larger than their intra-taxon distances compared with the other species of *Hypolepis* and the ratio between the minimum inter-taxon distance and the maximum intra-taxon distance is 11 (Fig. 3).

## Discussion

*Hypolepis robusta* was first reported by Chu (1992), being endemic to the Yunnan Province (Chu et al. 2006). After carefully comparing the type (including holotype and lectotype) of *H. robusta* and *H. alpina*, it was found that their morphological characteristics, e.g. the adventitious bud at stipe base, frond size, indusium and others (lamina, stipe, hair), are basically the same.

**Figure 4.**
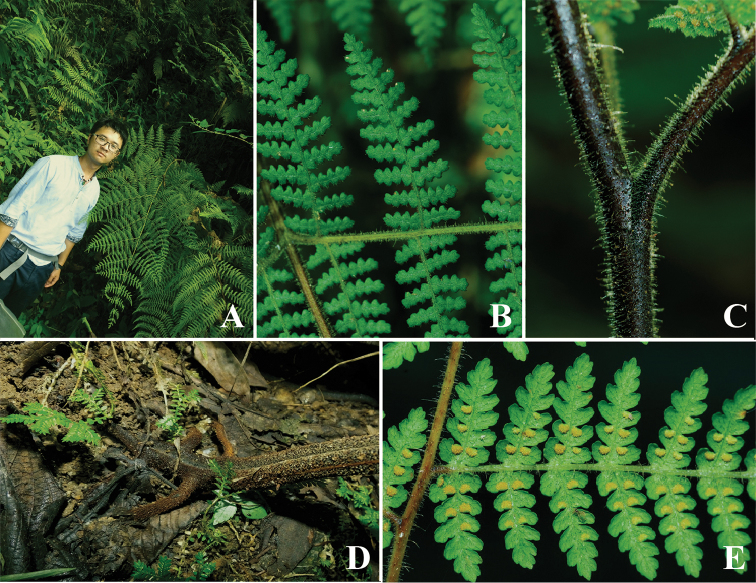
*Hypolepis alpina*. **A** Frond size (photographed by H. Shang in Fugong) **B** Lamina (photographed by R. Knapp in Nantou) **C** Hair (photographed by R. Knapp in Nantou) **D** The adventitious bud at stipe base (photographed by H. Shang in Fugong) **E** Indusium (photographed by R. Knapp in Nantou).

One of the main differences of *H. robusta* and *H. alpina* (*H. alte-gracillima*), mentioned in the key in *Flora Yunnanica*, is that the former has a few adventitious buds growing on both sides of the stipe base (Chu et al. 2006). However, when several specimens were examined in the herbarium and those from the authors’ own collection, it was found that *H. alpina* also has this feature (Fig. 4D). Therefore, it is concluded that the character used in the description is not relevant for distinguishing between *H. robusta* and *H. alpina*. Moreover, other Asian species of *Hypolepis* also develop adventitious buds, such as *H. pallida* (Blume) Hook. and *H. tenuifolia* (G. Forster) Bernhardi.

Another character used to support *H. robusta* as a new species was its larger size than *H. alpina*. The latter was reported at higher altitudes in the Malaysian region, from about 1,500–3,500 m and also as low as 1,100 m on Mt Kinabalu in Borneo (Brownsey 1987). However, there is considerable variation between plants from the highest elevations in New Guinea, which have rather smaller fronds and a dense covering of chestnut-brown non-glandular hairs, to those at lower altitudes in the northern part of its range (notably Taiwan), which have large fronds and very few chestnut hairs (Brownsey 1987). According to the description in *Flora Yunnanica*, *H. robusta* has a little larger frond than *H. alpina* (*H. alte-gracillima*). The field observation showed that *H. robusta* always occurs at altitudes about 1,000 m or even lower (Fig. 4A) and this is in accordance with the correlation between the altitudes and frond sizes mentioned in previous literature.

The characters of the indusium have been widely used in fern taxonomy. According to the previous literature of *H. alpina* and *H. robusta* (Brownsey 1987, Chu et al. 2006), they could be distinguished morphologically as follows: *H. robusta* has white indusium with marginal laceration, but *H. alpina* has a reflexed broad green lamina flap. Based on careful observations of all available material, it was found that their indusia are both half membranaceous at the margins and still green at the base (Fig. 4E). However, when the sori mature, the membranaceous margin becomes lacerated or exfoliated and the base can lose its chlorophyll, thus turning white. This difference may therefore be due to the fact that the descriptions have been made at different periods for the same species, a fact which had been previously ignored.

In addition to the morphological identification, a molecular phylogenetic analysis was also undertaken. The phylogenetic analysis of the *rbcL* and *trnL-F* sequences strongly supported the monophyly of *H. alpina* and *H. robusta* as a phylogenetic species with a wide distribution and distantly related to *H. polypodioides* (Fig. 2). The DNA barcoding analysis based on the K2P model revealed a significant gap between the inter-taxon and intra-taxon genetic distances, the distance in the *H. robusta* and *H. alpina* clade range from zero to 0.002, which is much lower than the inter-taxon distance and, in particular, the genetic distance between the *H. alpina* from Taiwan and the *H. robusta* from its type locality in Yunnan is zero (Fig. 3).

To sum up, not only does the morphological comparison identify *H. robusta* and *H. alpina* as conspecies, but also the phylogeny analysis identifies these as conspecies. Therefore, *H. robusta* is here reduced to a synonym of *H. alpina*. Consequently, *H. alpina* has three new distribution records in Guangdong, Guangxi and Hainan Island of China (Fig. 1). The new distribution records of *H. alpina* fill in gaps of the disjunct distribution defined in previous studies.

### Taxonomic treatment

#### Hypolepis alpina

Taxon classificationPlantaePolypodialesDennstaedtiaceae

(Blume) Hook. (1852: 63)

Hypolepis alpina (Blume) Hook. (1852: 63). Cheilanthes alpina Blume (1828: 138). Cheilanthes dissecta Hook. & Arn. (1841: 75). Hypolepis dissecta (Hook. & Arn.) Brack. (1854: 89–90). Hypolepis alte-gracillimaHayata (1915: 295–297).
Type: Indonesia. Java: Jawa Barat, Gede, *Blume C. L.* (Lectotype: L-0051753!, L-0051754!).

##### Type.

China. Yunnan: Fugong County, 1980, *W. M. Chu* (Holotype: PYU-01017821!, PYU-01017822!, PYU-01017823!, PYU-01017824!).

Fronds up to 1.7 m high. Rhizome long-creeping, 2–10 mm diameter, densely covered in red-brown hairs up to 3 mm long. Stipes reddish-brown, 12–70 cm long, 1.5–13 mm diameter, grooved adaxially, covered in red-brown non-glandular hairs up to 2 mm long and shorter glandular hairs, few adventitious buds at both sides of the stipe base; lamina ovate in outline, 3– or 4–pinnate, 20–80 (–130) cm × 10–90 cm, rachis red-brown or chestnut-brown at base, becoming chestnut-brown or yellow-brown at apex, densely covered in red-brown or chestnut-brown glandular hairs up to 0.5 mm long with occasional much longer non-glandular hairs; primary pinnae 15–30 pairs, opposite or sub-opposite, the largest at or near base, ovate to narrowly triangular, 10–52 cm × 3–28 cm; secondary pinnules narrowly ovate to ovate, 2–14 cm × 0.8–5 cm; ultimate pinnules to 10 mm × 5 mm. Sori circular or ovate, originating away from margins, without hairs between sporangia, protected by reflexed adaxial indusium, green at base and half membranaceous at margin, when the sori turn mature, the membranaceous margin becomes lacerated or exfoliated and the base part may turn white. Spores very pale under light microscope, perispores with interconnecting flattened projections, (32–) 34–37 (–40) µm × (20–) 21–25 (–28) μm.

##### Distribution.

China (Guangdong, Guangxi, Hainan, Taiwan, Yunnan), Indonesia, Japan, Malaysia, Papua New Guinea, Philippines.

## Supplementary Material

XML Treatment for Hypolepis alpina

## Supplementary materials

Supplementary material 1 Table S1 Data type: (measurement/occurence/multimedia/etc.) Explanation note: Herbarium specimens information of Hypolepis alpina and Hypolepis robusta samples checked in this study. This is an open access article distributed under the terms of the Creative Commons Attribution License (CC BY 4.0), which permits unrestricted use, distribution, and reproduction in any medium, provided the original author and source are credited.Morigengaowa, Jun-Jie Luo, Ralf Knapp, Hong-Jin Wei, Bao-Dong Liu, Yue-Hong Yan, Hui Shang
