# Supplementary material for: The identity of Hypolepis robusta, as a new synonym of Hypolepis alpina (Dennstaedtiaceae), based on morphology and DNA barcoding and the new distribution
Source: PhytoKeys. 2018 Mar 15;(96):35–45. doi: 10.3897/phytokeys.96.23470 (PMC6283266; doi:10.3897/phytokeys.96.23470)
Supplement: Supplementary material 1 — Table S1 [file phytokeys-96-035-s001.doc]

**Supplementary material Table S1. Herbarium specimens information of *Hypolepis alpina* and *Hypolepis robusta* samples checked in this study .**

| **No.** | **Name on Label** | **Collector** | **Voucher** | **Locality** | **Collection Date** | **Herbarium** |
| --- | --- | --- | --- | --- | --- | --- |
| 1 | *Hypolepis* alpina (Blume) Hook | Blume C. L. | s.n. | Gede, Jawa Barat, Java, Indonesia | NA | L |
| 2 | *Hypolepis* alpina (Blume) Hook | Blume C. L. | s.n. | Gede, Jawa Barat, Java, Indonesia | NA | L |
| 3 | [*Hypolepis robusta* W. M. Chu](http://www.nsii.org.cn/2017/species.php?lname=Hypolepis robusta) | Chu W. M. | 11577-1’ | Maji Village, Fugong County, Yunnan, China | 1980.10.05 | PYU |
| 4 | [*Hypolepis robusta* W. M. Chu](http://www.nsii.org.cn/2017/species.php?lname=Hypolepis robusta) | Chu W. M. | 11577-2’ | Maji Village, Fugong County, Yunnan, China | 1980.10.05 | PYU |
| 5 | [*Hypolepis robusta* W. M. Chu](http://www.nsii.org.cn/2017/species.php?lname=Hypolepis robusta) | Chu W. M. | 11577-3’ | Maji Village, Fugong County, Yunnan, China | 1980.10.05 | PYU |
| 6 | [*Hypolepis robusta* W. M. Chu](http://www.nsii.org.cn/2017/species.php?lname=Hypolepis robusta) | Chu W. M. | 11577-1 | Maji Village, Fugong County, Yunnan, China | 1980.10.06 | PYU |
| 7 | *Hypolepis* alpina (Blume) Hook | Croft J. R.. | LAE61699 | Port Moresby sub-dist., Victoria Range, Central S.E. slope to Mt., Papua New Guinea | 1974.07.07 | K |
| 8 | *Hypolepis* alpina (Blume) Hook | Croxall J.P.; Parris B. S. | 6324 | NW side, Southern Highlands Mt Giluwe, Papua New Guinea, | 1997.05.22 | K |
| 9 | *Hypolepis* alpina (Blume) Hook | Fleischer M. | s.n. | Tjibodes am Gedeh, Java, Indonesia | 1902.01 | US |
| 10 | *Hypolepis* alpina (Blume) Hook | Hoogland R. D. | 9935 | Salasaket Rangem Huon Peninsula, Gimdoh, Morobe, Papua New Guinea | 1964.9.25 | US |
| 11 | *Hypolepis* alpina (Blume) Hook | Hoogland R. D. | 9935 | Salasaket Rangem Huon Peninsula, Gimdoh, Morobe, Papua New Guinea | 1964.9.25 | US |
| 12 | [*Hypolepis robusta* W. M. Chu](http://www.nsii.org.cn/2017/species.php?lname=Hypolepis robusta) | Wei H. J.; Gu Y. F.; Zhu X. F. | DRS005 | Darong Mountain, Guangxi, China | 2013.07.19 | CSH |
| 13 | *Hypolepis* alpina (Blume) Hook | [Knapp R..](https://science.mnhn.fr/all/list?recordedBy=Knapp, R.) | Knapp 1796 | WuLai, XinBei City, Taiwan, China | 2009.03.15 | P |
| 14 | *Hypolepis* alpina (Blume) Hook | [Knapp R..](https://science.mnhn.fr/all/list?recordedBy=Knapp, R.) | Knapp 1796 | WuLai, XinBei City, Taiwan, China | 2009.03.15 | P |
| 15 | *Hypolepis* alpina (Blume) Hook | [Knapp R..](https://science.mnhn.fr/all/list?recordedBy=Knapp, R.) | Knapp 1796 | WuLai, XinBei City, Taiwan, China | 2009.03.15 | P |
| 16 | *Hypolepis* alpina (Blume) Hook | [Knapp R..](https://science.mnhn.fr/all/list?recordedBy=Knapp, R.) | Knapp 1796 | WuLai, XinBei City, Taiwan, China | 2009.03.15 | P |
| 17 | *Hypolepis* alpina (Blume) Hook | [Knapp R..](https://science.mnhn.fr/all/list?recordedBy=Knapp, R.) | Knapp 1796 | WuLai, XinBei City, Taiwan, China | 2009.03.15 | P |
| 18 | *Hypolepis* alpina (Blume) Hook | [Knapp R..](https://science.mnhn.fr/all/list?recordedBy=Knapp, R.) | Knapp 1796 | WuLai, XinBei City, Taiwan, China | 2009.03.15 | P |
| 19 | *Hypolepis* alpina (Blume) Hook | [Knapp R..](https://science.mnhn.fr/all/list?recordedBy=Knapp, R.) | Knapp 1796 | WuLai, XinBei City, Taiwan, China | 2009.03.15 | P |
| 20 | *Hypolepis* alpina (Blume) Hook | [Knapp R..](https://science.mnhn.fr/all/list?recordedBy=Knapp, R.) | Knapp 1796 | WuLai, XinBei City, Taiwan, China | 2009.03.15 | P |
| 21 | *Hypolepis* alpina (Blume) Hook | [Knapp R..](https://science.mnhn.fr/all/list?recordedBy=Knapp, R.) | Knapp 3210 | WuLai, XinBei City, Taiwan, China | 2013.08.24 | P |
| 22 | *Hypolepis* alpina (Blume) Hook | [Knapp R..](https://science.mnhn.fr/all/list?recordedBy=Knapp, R.) | Knapp 3210 | WuLai, XinBei City, Taiwan, China | 2013.08.24 | P |
| 23 | *Hypolepis* alpina (Blume) Hook | [Knapp R..](https://science.mnhn.fr/all/list?recordedBy=Knapp, R.) | Knapp 3210 | WuLai, XinBei City, Taiwan, China | 2013.08.24 | P |
| 24 | *Hypolepis* alpina (Blume) Hook | [Knapp R..](https://science.mnhn.fr/all/list?recordedBy=Knapp, R.) | Knapp 1832 | WuLai, XinBei City, Taiwan, China | 2009.05.01 | P |
| 25 | *Hypolepis* alpina (Blume) Hook | Knapp R.. | Knapp 4486 | Yilan County, Taiwan, China | NA | P |
| 26 | *Hypolepis alte-gracillima* Hayata | Kuo C. M. | 2090 | Kuantaohsi, Nantou, Taiwan, China | 1972.09.06 | TAI |
| 27 | *Hypolepis* alpina (Blume) Hook | Li S. T. | 846 | Cangyuan County, Yunnan, China | 1980.08.03 | KUN |
| 28 | *Hypolepis alte-gracillima* Hayata | Matuda E. | s.n. | Wuweishan, Pingtung, Taiwan, China | 1917.03.30 | TAI |
| 29 | *Hypolepis alte-gracillima* Hayata | [Otomasu M.](http://www.cvh.ac.cn/spuser/344936) | 34331 | Prov. Ohsumi, Sata-mura, Hetuca, Japan | 1959.03.31 | PE |
| 30 | *Hypolepis* alpina (Blume) Hook | Qing S. D. | 7312 | Gongshan County, Yunnan, China | 1982.06.13 | KUN |
| 31 | *Hypolepis* alpina (Blume) Hook | Qing S. D. | 7312 | Gongshan County, Yunnan, China | 1982.06.13 | KUN |
| 32 | [*Hypolepis robusta* W. M. Chu](http://www.nsii.org.cn/2017/species.php?lname=Hypolepis robusta) | Shang H. | HND6 | Bawang mountain, Hainan, China | 2012.12.02 | CSH |
| 33 | [*Hypolepis robusta* W. M. Chu](http://www.nsii.org.cn/2017/species.php?lname=Hypolepis robusta) | Shang H.; Huang K. R. | SG0958 | Shengtang Mountain, Guangxi, China | 2015.09.18 | CSH |
| 34 | [*Hypolepis robusta* W. M. Chu](http://www.nsii.org.cn/2017/species.php?lname=Hypolepis robusta) | Shang H.; Luo J. J. | SG1812 | Ada Village, Fugong County, Yunnan, China | 2017.06.15 | CSH |
| 35 | *Hypolepis* alpina (Blume) Hook | Shang H.; Luo J. J. | SG1838 | Dulongjiang Village, Gongshan County, Yunnan, China | 2017.06.16 | CSH |
| 36 | *Hypolepis* alpina (Blume) Hook | Shang H.; Luo J. J. | SG1871 | Dulongjiang Village, Gongshan County, Yunnan, China | 2017.06.17 | CSH |
| 37 | [*Hypolepis robusta* W. M. Chu](http://www.nsii.org.cn/2017/species.php?lname=Hypolepis robusta) | Shang H. | HND5 | Bawang Mountain, Hainan, China | 2012.12.15 | CSH |
| 38 | [*Hypolepis robusta* W. M. Chu](http://www.nsii.org.cn/2017/species.php?lname=Hypolepis robusta) | Shang H. | SG2630 | Bulang Mountain, Menghai County, Yunnan, China | 2015.12.18 | CSH |
| 39 | [*Hypolepis robusta* W. M. Chu](http://www.nsii.org.cn/2017/species.php?lname=Hypolepis robusta) | Shang H.; Huang R. K. | SG0945 | Shengtang Mountain, Laibin City, Guangxi, China | 2015.09.18 | CSH |
| 40 | *Hypolepis* alpina (Blume) Hook | Shang H.; Luo J. J | SG1875 | Dulongjiang Vilage, Gongshan County, Yunnan, China | 2017.06.17 | CSH |
| 41 | *Hypolepis* alpina (Blume) Hook | Shang H.; Luo J. J. | SG1873 | Dulongjiang Vilage, Gongshan County, Yunnan, China | 2017.06.17 | CSH |
| 42 | *Hypolepis alte-gracillima* Hayata | Takeo Ito | s.n. | Arisan, Heisyana, Taiwan, China | 1914.05.31 | TAIF |
| 43 | *Hypolepis alte-gracillima* Hayata | Takeo Ito | s.n. | NA | NA | TAIF |
| 44 | *Hypolepis* alpina (Blume) Hook | Taku Miyazaki | 701119 | Kouchi Pref., Takaoka District, Yatabe Nakatosa Town, Japan | 2007.06.03 | PE |
| 45 | *Hypolepis* alpina (Blume) Hook | Taku Miyazaki | 701119 | Kouchi Pref., Takaoka District, Yatabe Nakatosa Town, Japan | 2007.06.03 | PE |
| 46 | *Hypolepis* alpina (Blume) Hook | Taku Miyazaki | 701026 | Kouchi Pref., Takaoka District, Nakatosa Town, Ohkawauchi Kaminokae, Japan | 2006.12.29 | PE |
| 47 | *Hypolepis* alpina (Blume) Hook | Taku Miyazaki | 701026 | Kouchi Pref., Takaoka District, Nakatosa Town, Ohkawauchi Kaminokae, Japan | 2006.12.29 | PE |
| 48 | *Hypolepis* alpina (Blume) Hook | Taku Miyazaki | 701119 | Kouchi Pref., Takaoka District, Yatabe Nakatosa Town, Japan | 2007.06.03 | PE |
| 49 | *Hypolepis* alpina (Blume) Hook | Taku Miyazaki | 601027 | Kochi Pref., Takaoka Distr, Kubokawa Town, Shiwa, Japan | 2005.12.31 | PE |
| 50 | *Hypolepis* alpina (Blume) Hook | Wu S. G. | 4099 | Maguan County, Yunnan, China | 1962.09.21 | KUN |
| 51 | *Hypolepis* alpina (Blume) Hook | Yan Y. H.; He Z. X. | YYH11628 | Xitou Village, Nantou County, Taiwan, China | 2013.05 | CSH |
| 52 | [*Hypolepis robusta* W. M. Chu](http://www.nsii.org.cn/2017/species.php?lname=Hypolepis robusta) | Yan Y. H.; He Z. X. | YYH12064 | Mengsong Village, Jinghong City, Yunnan, China | 2014.05 | CSH |
| 53 | [*Hypolepis robusta* W. M. Chu](http://www.nsii.org.cn/2017/species.php?lname=Hypolepis robusta) | Yan Y. H.; Xiang J. Y.; Morigengaowa | CFH09000280 | Dawei Mountain, Pingbian County, Yunnan, China | 2017.09.28 | CSH |
| 54 | *Hypolepis* alpina (Blume) Hook | Yan Y. H.; He Z. X. | YYH11629 | Xitou Village, Nantou County, Taiwan, China | 2013.05 | CSH |
| 55 | [*Hypolepis robusta* W. M. Chu](http://www.nsii.org.cn/2017/species.php?lname=Hypolepis robusta) | Yan Y. H.; Wei H. J.; Wang Y.; Zhu X. F. | WYD558 | Dawu Mountain, Xinyi County, Maoming City, Guangdong, China | 2014.08.14 | CSH |
| 56 | [*Hypolepis robusta* W. M. Chu](http://www.nsii.org.cn/2017/species.php?lname=Hypolepis robusta) | Yan Y. H.; Wei H. J.; Wang Y.; Zhu X. F. | WYD421 | Dawu Mountain, Xinyi County, Maoming City, Guangdong, China | 2014.08.14 | CSH |
| 57 | [*Hypolepis robusta* W. M. Chu](http://www.nsii.org.cn/2017/species.php?lname=Hypolepis robusta) | Yan Y. H.; Wei H. J.; Wang Y.; Zhu X. F. | WYD498 | Dawu Mountain, Xinyi County, Maoming City, Guangdong, China | 2014.08.14 | CSH |
| 58 | [*Hypolepis robusta* W. M. Chu](http://www.nsii.org.cn/2017/species.php?lname=Hypolepis robusta) | Yan Y. H.; Wei H. J.; Wang Y.; Zhu X. F. | WYD455 | Dawu Mountain, Xinyi County, Maoming City, Guangdong, China | 2014.08.14 | CSH |
| 59 | [*Hypolepis robusta* W. M. Chu](http://www.nsii.org.cn/2017/species.php?lname=Hypolepis robusta) | Yan Y. H.; Wei H. J.; Wang Y.; Zhu X. F. | WYD498 | Dawu Mountain, Xinyi County, Maoming City, Guangdong, China | 2014.08.14 | CSH |
| 60 | [*Hypolepis robusta* W. M. Chu](http://www.nsii.org.cn/2017/species.php?lname=Hypolepis robusta) | Yan Y. H.; Wei H. J.; Wang Y.; Zhu X. F. | WYD569 | Dawu Mountain, Xinyi County, Maoming City, Guangdong, China | 2014.08.14 | CSH |
| 61 | [*Hypolepis robusta* W. M. Chu](http://www.nsii.org.cn/2017/species.php?lname=Hypolepis robusta) | Yan Y. H.; Wei H. J.; Wang Y.; Zhu X. F. | WYD275 | Dawu Mountain, Xinyi County, Maoming City, Guangdong, China | 2014.08.14 | CSH |
| 62 | [*Hypolepis robusta* W. M. Chu](http://www.nsii.org.cn/2017/species.php?lname=Hypolepis robusta) | Yan Y. H.; Wei H. J.; Wang Y.; Zhu X. F. | WYD276 | Dawu Mountain, Xinyi County, Maoming City, Guangdong, China | 2014.08.14 | CSH |
| 63 | [*Hypolepis robusta* W. M. Chu](http://www.nsii.org.cn/2017/species.php?lname=Hypolepis robusta) | Yan Y. H.; Wei H. J.; Wang Y.; Zhu X. F. | WYD345 | Dawu Mountain, Xinyi County, Maoming City, Guangdong, China | 2014.08.14 | CSH |
| 64 | [*Hypolepis robusta* W. M. Chu](http://www.nsii.org.cn/2017/species.php?lname=Hypolepis robusta) | Yan Y. H.; Wei H. J.; Wang Y.; Zhu X. F. | WYD569 | Dawu Mountain, Xinyi County, Maoming City, Guangdong, China | 2014.08.14 | CSH |
| 65 | [*Hypolepis robusta* W. M. Chu](http://www.nsii.org.cn/2017/species.php?lname=Hypolepis robusta) | Yan Y. H.; Wei H. J.; Wang Y.; Zhu X. F. | WYD574 | Dawu Mountain, Guangdong, China | 2014.08.14 | CSH |
| 66 | [*Hypolepis robusta* W. M. Chu](http://www.nsii.org.cn/2017/species.php?lname=Hypolepis robusta) | Zhang X. C.; Guo Z. Y.; Sui Y. M.; Wei H. J. | ZXC8469 | Gulinqing Village, Maguan County, Yunnan, China | 2017.04.11 | CSH |
| 67 | [*Hypolepis robusta* W. M. Chu](http://www.nsii.org.cn/2017/species.php?lname=Hypolepis robusta) | Zhang X. C.; Guo Z. Y.; Sui Y. M.; Wei H. J. | ZXC8465 | Gulinqing Village, Maguan County, Yunnan,China | 2017.04.11 | CSH |
| 68 | [*Hypolepis robusta* W. M. Chu](http://www.nsii.org.cn/2017/species.php?lname=Hypolepis robusta) | Zhou X. L.; Zhang J. B. | ZXL05763 | Mengsong Village, Jinghong City, Yunnan, China | 2015.10.09 | CSH |
| 69 | [*Hypolepis robusta* W. M. Chu](http://www.nsii.org.cn/2017/species.php?lname=Hypolepis robusta) | Zhou X. L.; Zhang J. B. | ZXL05765 | Mengsong Village, Jinghong City, Yunnan, China | 2015.10.09 | CSH |

*Note* : NA = not available. Table order is sorted by collector.
